# Potential of Trap Crops for Integrated Management of the Tropical Armyworm, *Spodoptera litura* in Tobacco

**DOI:** 10.1673/031.010.11701

**Published:** 2010-07-26

**Authors:** Zhongshi Zhou, Zepeng Chen, Zaifu Xu

**Affiliations:** ^1^ Department of Entomology, College of Nature Resources and Environment, South China Agricultural University, Guangzhou, 510642, The People's Republic of China; ^2^ State Key Laboratory for Biology of Plant Diseases and Insect Pests, Institute of Plant Protection, Chinese Academy of Agricultural Sciences, Center for Management of Invasive Alien Species, Ministry of Agriculture, P. R. China, Beijing 100193, The People's Republic of China; ^3^ Guangdong Company of Tobacco, Guangzhou, 510030, The People's Republic of China

**Keywords:** *Colocasia esculenta*, cultural control, trap crop, oviposition preference, attraction

## Abstract

The tropical armyworm, *Spodoptera litura* (F.) (Lepidoptera: Noctuidae), is an important pest of tobacco, *Nicotiana tabacum* L. (Solanales: Solanaceae), in South China that is becoming increasingly resistant to pesticides. Six potential trap crops were evaluated to control *S. litura* on tobacco. Castor bean, *Ricinus communis* L. (Malpighiales: Euphorbiaceae), and taro, *Colocasia esculenta* (L.) Schott (Alismatales: Araceae), hosted significantly more *S. litura* than peanut, *Arachis hypogaea* L. (Fabales: Fabaceae), sweet potato, *Ipomoea batata* Lam. (Solanales: Convolvulaceae) or tobacoo in a greenhouse trial, and tobacco field plots with taro rows hosted significantly fewer *S. litura* than those with rows of other trap crops or without trap crops, provided the taro was in a fast-growing stage. When these crops were grown along with eggplant, *Solanum melongena* L. (Solanales: Solanaceae), and soybean, *Glycines max* L. (Fabales: Fabaceae), in separate plots in a randomized matrix, tobacco plots hosted more *S. litura* than the other crop plots early in the season, but late in the season, taro plots hosted significantly more *S. litura* than tobacco, soybean, sweet potato, peanut or eggplant plots. In addition, higher rates of *S. litura* parasitism by *Microplitis prodeniae* Rao and Chandry (Hymenoptera: Bracondidae) and *Campoletis chlorideae* Uchida (Ichnumonidae) were observed in taro plots compared to other crop plots. Although taro was an effective trap crop for managing *S. litura* on tobacco, it did not attract *S. litura* in the seedling stage, indicating that taro should either be planted 20–30 days before tobacco, or alternative control methods should be employed during the seedling stage.

## Introduction

The tropical armyworm, *Spodoptera litura* (F.) (Lepidoptera: Noctuidae) is a generalist herbivore and an important pest in many agricultural cropping systems. Wu et al. (2004) reported that *S. litura* infested more than 290 species of plants belonging to 99 families. In South China, *S. litura* is an important insect pest of tobacco causing serious defoliation and its management can be difficult. Chemical control is a popular management tactic (Peter and David 1988; Kumar and Parmar 1996), but this has led to many problems, e.g. the resistance of *S. litura* to traditional insecticides, environmental pollution, human health impacts, and injury to beneficial species, etc. (Kranthi et al. 2002). Management failures of *S. litura* have become common in Southeast Asia, India and China, necessitating development of novel control methods, especially in tobacco (Jayanthi and Padmavathamma 2001; Zhou and Huang 2002).

There has been a resurgence of interest in trap crops recently because of concerns about the many negative effects of pesticides (Barari et al. 2005; Khan et al. 2006). Trap cropping is an alternative method of control in which plants are deployed to attract, intercept, retain and/or reduce targeted insects or the pathogens they vector in order to reduce damage to the cash crop (Shelton and Badenes-Perez 2006). The effectiveness of any trap cropping system depends on an interplay between the spatial arrangement of the trap crop system and pest population processes, such as movement and reproduction (Hannunen 2005; Cárcamo et al. 2007). Trap crops have been used successfully to manipulate the behavior of herbivores and reduce pest pressure (Hartwig 2002; Gbèhounou and Adango 2003). Many studies have revealed variation in the developmental capability and host preference of *S. litura* among crops (Singh and Byas 1975; Balasubramanian et al. 1984; Chhibber *et al.* 1985). Early studies indicated that castor, *Ricinus communis* was a highly suitable host plant (Balasubramanian *et al.* 1984; Chibber et al. 1985), leading to speculation that castor might be used as a trap crop to attract and destroy *S. litura.*

Natural enemies (e.g. parasitoids) can significantly reduce the population densities of pests and trap crop systems are compatible with biological control, sometimes even improving it (Williams 2004). For instance, green manure plants such as *Sesbania roxburghii* can provide parasitoids with both refuges and nectar, increasing rates of parasitism on sugarcane pests when intercropped with sugarcane in the field (Pu 1978). Andow (1991) and Khan et al. (1997) both concluded that trap crop systems often hosted more parasitoids when compared with simple monocultures.

These experiments investigated the oviposition preference of *S. litura* on selected host crops in a greenhouse, the population densities of *S. litura* larvae in field plots of the different crop plants, and rates of parasitism by two important larval parasitoids, *Microplitis prodeniae* Rao and Chandry (Hymenoptera: Braconidae) and *Campoletis* chlorideae Uchida (Hymenoptera: Ichneumonidae). The objective was to select an effective trap crop for managing this pest on tobacco.

## Materials and Methods

### Oviposition preferences of *S. litura* in a greenhouse

This experiment was conducted in the greenhouse of Guangxi University, Nanning City, in the Zhuang Autonomous Region of Guangxi, in 2005. The attractiveness of castor, *Ricinus communis* L. (Malpighiales: Euphorbiaceae), taro, *Colocasia esculenta* (L.) Schott (Alismatales: Araceae), peanut, *Arachis hypogaea* L. (Fabales: Fabaceae), and sweet potato, *Ipomoea batata* (L.) Lam. (Solanales: Convolvulaceae) was tested relative to tobacco, *Nicotiana tabacum* L. (Solanales: Solanaceae). Healthy seedlings of the five crops were transplanted into 35 cm diameter plastic pots with uniform soils with N: P: K fertilizer (13: 7:15) added to maintain normal plant growth. These plants were tested in the seedling stage and in stages of rapid vegetative growth, i.e., 10 days and 50 days after transplanting, respectively. One plant of each type was placed in a large cage made of mesh (1.2 m × 1.2 m × 1.5 m) and then 10 pairs of virgin adults of *S. litura* were released in the cage. The experiment was replicated three times. The number of egg masses on each plant was recorded daily for seven days after moth release.

**Figure 1.**
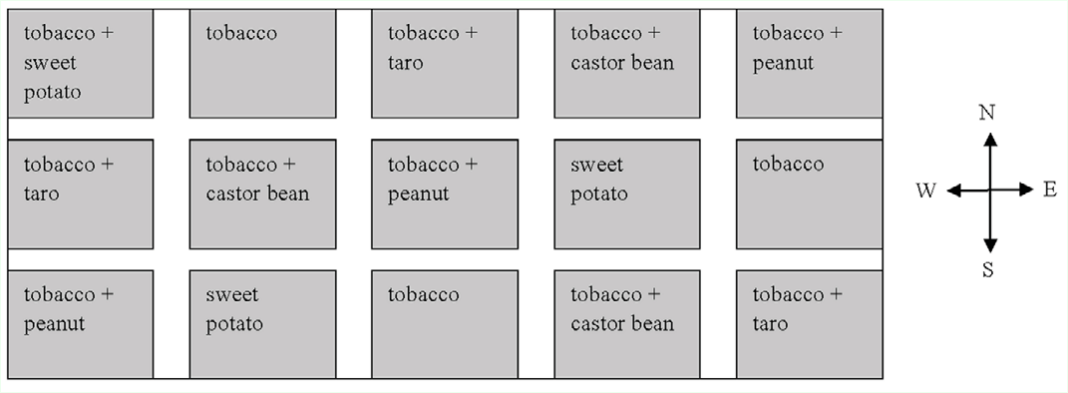
Layout of plots in the 2005 field trial in. Plots were randomly assigned to one of five treatments where tobacco was interplanted with two rows of one of four trap crops. High quality figures are available online.

### Oviposition preferences of *S. litura* inoculated in field plots

This experiment was conducted at the experimental farm of Guangxi University in 2005 in an experimental field of about 0.3 ha. The experiment had five treatments with three replications, producing fifteen plots of about 0.01 ha each in a completely randomized design (Figure 1). Adjacent plots were separated by one row of bare soil (10 m long from south to north and 2 m wide from east to west). Twelve rows of tobacco and four rows of the trap crop were planted in each plot, with one row of trap crop (either castor, taro, peanut or sweet potato) planted every four rows of tobacco in each intercropped plot, and tobacco only in control plots. There were 22 plants in each row of tobacco and taro, 18 plants in each castor row, and 100 plants in each row of peanut and sweet potato. When crops reached a stage of rapid vegetative growth, 20 small holes (5 cm diameter × 3 cm deep) were dug in each plot and *S. litura* pupae (10 male and 10 female) were placed in the holes and covered with soil. Surveys were conducted on the second, fourth and seventh day after placement of pupae by randomly sampling 80 tobacco plants and 20 trap crop plants in each plot and recording the number of egg masses on them.

### Oviposition preferences of *S. litura* naturally infesting field plots

**Figure 2.**
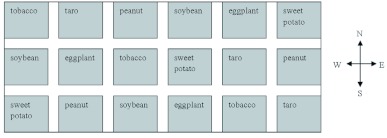
Layout of plots for the naturally-infested field trial in 2006. Plots were randomly assigned to one of six treatments, each planted with a single crop type. High quality figures are available online.

This experiment was conducted at the experimental farm of the Nanxiong Research Institute of Tobacco, Nanxiong City, Guangdong Province in 2006. In this case, a natural population of *S. litura* infested the experiment from an adjacent infested field. The study field was about 0.36 ha and was divided into 18 randomized plots of about 0.01 ha each. Six treatments were employed, each with three replications (Figure 2). Adjacent plots were again separated by one row of bare soil (as above). Taro, peanut, sweet potato, tobacco, soybean (*Glycine max* L.) and eggplant (*Solanum melongena* L.) were planted in the various plots on 20 February and the population dynamics of *S. litura* larvae were monitored from 17 April to 21 June in 2006. When the first generation of *S. litura* in the adjacent field began emerging, the study field was sampled every five days by counting the number of larvae on 100 plants of tobacco in each plot at each survey stage. In addition, the number of egg masses in each crop plot was recorded bimonthly, from March to June in 2006.

### Rates of *S. litura* parasitism by *M. prodeniae* and *C. chlorideae* in naturally infested field plots

Random samples of second generation of *S. litura* were obtained every six days from 12 May to 17 June in the naturally infested field plots in 2006. Eighty second instar larvae were collected from each crop plot on each survey date and taken to laboratory where they were reared out on fresh tobacco leaves provided fresh daily until emergence of moths or parasitoid adults.

### Statistical analysis

All data were checked for normality and homoscedasticity and were log10 (x+1) transformed when necessary. A one-way ANOVA was conducted to test for effects of treatment and means were separated with Fisher's protected LSD (α = 0.05).

## Results

### Oviposition preferences of *S. litura* in a greenhouse

There was no difference among crop types in the number of *S. litura* egg masses on plants in the seedling stage (*F* = 2.06; df = 4; *P* = 0.162), but significant differences emerged when crops entered stages of rapid vegetative growth (*F* = 114.53; df = 4; *P* < 0.0001), with taro and castor plants hosting significant numbers of *S. litura* egg masses before any were laid on other crop types (Table 1).

### Oviposition preferences of *S. litura* inoculated in field plots

**Figure 3.**
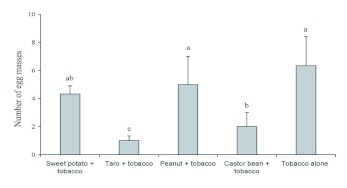
Mean (± SEM) cumulative numbers of *Spodoptera litura* egg masses (data pooled from three surveys of 80 plants/plot) on tobacco plants in plots with different trap crop rows in a field trial where pupae of *S. litura* were inoculated in the soil. Histograms bearing the same letters were not significantly different (one-way ANOVA followed by Fisher's LSD, α = 0.05). High quality figures are available online.

**Figure 4.**
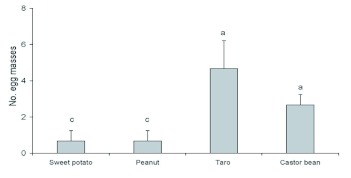
Mean cumulative numbers of egg masses (± SEM) on four trap crops when interplanted with tobacco in different plots in 2005 (data from three surveys pooled). Histograms bearing the same letters were not significantly different (one-way ANOVA followed by Fisher's LSD test, α = 0.05). High quality figures are available online.

There was a significant effect of trap crop treatment on the number of *S. litura* egg masses on tobacco plants (*F* = 7.45, df = 4; *P* = 0.0048). Tobacco plants in plots with taro or castor rows hosted significantly fewer egg masses than those in plots with sweet potato or peanut rows, or in plots of pure tobacco (Figure 3). In addition, taro plants hosted more *S. litura* egg masses compared with castor bean, which in turn hosted more than either sweet potato or peanut (F = 15.73; df = 3; *P* = 0.001; Figure 4).

### Oviposition preferences of *S. litura* naturally infesting field plots

**Figure 5.**
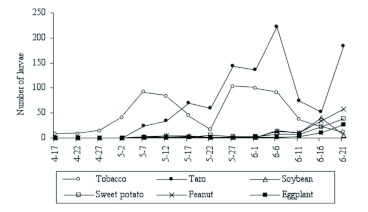
Mean total numbers of *Spodoptera litura* larvae observed per 100 plants (average of three plots) in monocultured plots of six crop species in 2006. High quality figures are available online.

**Figure 6.**
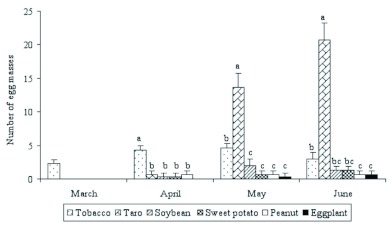
Mean numbers of *Spodoptera litura* egg masses per 100 plants in field plots of six different crops in 2006 (cumulative data for two surveys). Histograms bearing the same letters were not significantly different within months (one-way ANOVA followed by Fisher's LSD, α = 0.05). High quality figures are available online.

Population densities of *S. litura* larvae were highest in tobacco plots early in the season, but late in the season, densities in taro plots were the highest among all crop types (Figure 5). In March and April, when crops were in the seedling stage, tobacco hosted more *S. litura* than any other crop (March: *F* = 49.00; df=5; *P*<0.0001 and April: *F* = 28.52, df = 5; *P* < 0.0001). In May and June, when crops were rapidly growing, their relative attractiveness to *S. litura* changed. In May, *S. litura* oviposition preference was taro > tobacco > all other crops (*F* = 71.76; df = 5; *P* < 0.0001), whereas in June it was taro > tobacco (*F* = 130.01; df = 5; *P* < 0.0001; Figure 6).

### Rates of *S. litura* parasitism in naturally infested field plots

**Figure 7.**
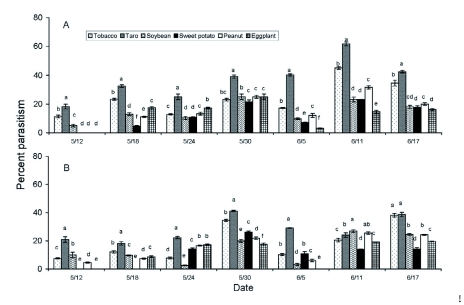
Parasitism rates of *Spodoptera litura* larvae by *Microplitis prodeniae* (A) and *Campoletis chlorideae* (B) across seven sampling dates in replicated plots of six different crops in 2006. On each date, 100 plants were sampled in each of three plots of each crop type. Histograms bearing the same letter were not significantly different (one-way ANOVA followed by Fisher's LSD, α = 0.05) within sampling dates. High quality figures are available online.

Rates of *S. litura* parasitism by *M. prodeniae* and *C. chlorideae* differed significantly among the six crops over the course of our experiment (Figure 7). *Spodoptera litura* larvae suffered higher rates of parasitism by *M. prodeniae* on taro plants than on other crops (*F* = 232.46; df = 5; *P* < 0.0001 on 12 May; *F* = 511.57; df = 5; *P* < 0.0001 on 18 May; *F* = 84.35; df = 5; *P* < 0.0001 on 24 May; *F* = 56.76; df = 5; *P* < 0.0001 on 30 May; *F* = 998.63; df = 5; *P* < 0.0001 on 5 June; *F* = 675.88; df = 5; *P* < 0.0001 on 11 June; *F* = 242.24; df = 5; *P* < 0.0001 on 17 June. Taro also resulted in higher rates of *S. litura* parasitism by *C. chlorideae* than did other crops on all dates except 11 June (*F* = 133.73; df = 5; *P* < 0.0001 on 12 May; *F* = 221.24; df = 5; *P* < 0.0001 on 18 May; *F* = 436.87; df = 5; *P* < 0.0001 on 24 May; *F* = 394.81; df = 5; *P* < 0.0001 on 30 May; *F* = 417.38; df = 5; *P* < 0.0001 on 5 June; *F* = 75.42; df = 5; *P* < 0.0001 on 11 June; *F* = 320.81; df = 5; *P* < 0.0001 on 17 June).

**Table 1.**
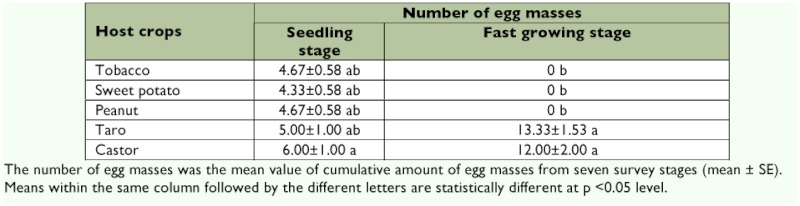
Oviposition by *S. litura* females on five host crops in each of two growth stages in a greenhouse (Mean±SE, three sampling dates pooled for each growth stage)

## Discussion

Trap cropping is an alternative method of pest control in which plants are deployed to attract, intercept and retain the damaging stages of insect pests in order to reduce damage to a cash crop (Michaud et al. 2007; Shelton et al. 2008). An effective trap crop must be significantly more attractive to an insect pest than the cash crop for a significant duration of the crop cycle. Furthermore, the costs associated with the trap crop must be less than the benefits of increased yield in the cash crop (Shelton and Badenes-Perez 2006; Michaud et al. 2007). In recent years, trap crops have received increasing attention as a possible pest management alternative. For example, Sequeira *et al.* (2001) evaluated the potential of various companion crops to divert *Helicoverpa* spp. from ovipositing on chickpea, *Cicer arietinum*, but none reduced infestation of chick pea to acceptable levels. Rousse *et al.* (2003) found that rows of turnips, *Brassica rapa*, interplanted with broccoli, *Brassica oleracea*, reduced damage by the cabbage maggot, *Delia radicum* largely because of increased rates of predation and parasitism by *Aleochara* spp. (Coleoptera: Staphylinidae). Michaud et al. (2007) demonstrated that perimeter rows of sunflower can effectively collect and retain ovipositing stem borers, *Dectes texanus* females and greatly reduce their infestation of soybean.

Previous studies had suggested that either castor bean or taro might be used as a trap crop to attract *S. litura* (Balasubramanian et al. 1984; Wu et al. 2004). In our greenhouse experiment, taro hosted significantly more *S. litura* egg masses than did the other four crops when in stages of rapid vegetative growth. In the field trial in 2005, tobacco plots interplanted with taro rows hosted significantly fewer *S. litura* egg masses than did the plots with rows of other trap crops or in pure stands of tobacco. Comparing the candidate traps crops, taro hosted more *S. litura* egg masses compared to castor bean, which in turn hosted more than sweet potato or peanut. In some cases, trap crops only attract the target pest at a specific growth stage (Hokkanen 1991). In the 2006 field trial with separate plots of each plant type, tobacco plots hosted more *S. litura* than the other five crops, including taro, early in the season when plants were in the seedling stage. However, later in the season when crops were in more advanced stages of vegetative growth, plots of taro hosted significantly more *S. litura* than did plots of other plant types, confirming the results obtained in 2005.

The parasitoids *M. prodeniae* and *C. chlorideae* both parasitized *S. litura* larvae at higher rates in taro plots than in plots of other crops. Zhou et al. (2007) found that parasitism of *S. litura* by *M. prodeniae* and *C. chlorideae* on taro and tobacco was density-dependent and the higher densities of *S. litura* on taro than on other crops may partly account for this result. Furthermore, taro also provided an alternative host for *M. prodeniae* in the form of larvae of *Theretra pinastrina* (Martyn) (Lepidoptera: Sphingidae). Many parasitoid species find their hosts by olfaction and may benefit from associative learning about insect-plant associations (Mange and Cortesero 1996; Iizuka and Takasu 1998). Since both insect pests and infested plants may emit volatile semiochemicals that cue parasitoid search (Turlings et al. 1995; Fukushima et al. 2001), heavily-infested taro plants may attract more *M. prodeniae* and *C. chlorideae* and thus increase rates of *S. litura* parasitism on adjacent tobacco plants.

Our experiments indicated that taro could serve as an effective trap crop for managing *S. litura* in tobacco fields, both by acting as an egg sink and by improving rates of larval parasitism. However, further research is needed to determine the optimum spatial configuration of the two crops and whether or not tobacco yield benefits will be sufficient to justify the loss of tobacco production in taro rows. Since taro does not attract *S. litura* in the seedling stage, taro should either be planted 20–30 days before tobacco, or alternative control methods employed during the seedling stage.
